# A novel approach to the discovery of anti-tumor pharmaceuticals: searching for activators of liponecrosis

**DOI:** 10.18632/oncotarget.6440

**Published:** 2015-11-30

**Authors:** Anthony Arlia-Ciommo, Veronika Svistkova, Sadaf Mohtashami, Vladimir I. Titorenko

**Affiliations:** ^1^ Department of Biology, Concordia University, Montreal, Quebec, Canada

**Keywords:** cancer, anti-cancer therapeutics, lipid metabolism, programmed cell death, liponecrosis, yeast

## Abstract

A recently conducted chemical genetic screen for pharmaceuticals that can extend longevity of the yeast *Saccharomyces cerevisiae* has identified lithocholic acid as a potent anti-aging molecule. It was found that this hydrophobic bile acid is also a selective anti-tumor chemical compound; it kills different types of cultured cancer cells if used at concentrations that do not compromise the viability of non-cancerous cells. These studies have revealed that yeast can be successfully used as a model organism for high-throughput screens aimed at the discovery of selectively acting anti-tumor small molecules. Two metabolic traits of rapidly proliferating fermenting yeast, namely aerobic glycolysis and lipogenesis, are known to be similar to those of cancer cells. The mechanisms underlying these key metabolic features of cancer cells and fermenting yeast have been established; such mechanisms are discussed in this review. We also suggest how a yeast-based chemical genetic screen can be used for the high-throughput development of selective anti-tumor pharmaceuticals that kill only cancer cells. This screen consists of searching for chemical compounds capable of increasing the abundance of membrane lipids enriched in unsaturated fatty acids that would therefore be toxic only to rapidly proliferating cells, such as cancer cells and fermenting yeast.

## INTRODUCTION

Many pharmaceuticals that are currently used or undergoing clinical evaluation for cancer therapy have been developed as modulators of certain metabolic processes in cancer cells. These processes include the following: (1) nucleotide synthesis; (2) amino acid metabolism; (3) aerobic glycolysis, which is also known as “the Warburg effect”; (4) lipogenesis, a de novo synthesis of bulk quantities of membrane lipids; (5) a lipolytic formation of fatty acids from monoacylglycerols; (6) mitochondrial transport and oxidation of fatty acids; (7) pentose phosphate pathway; and (8) mitochondrial tricarboxylic acid (TCA) cycle and electron transport chain (ETC) [1-10]. A body of evidence implies that at least two of these processes, aerobic glycolysis and lipogenesis, are common metabolic features of cancer cells and rapidly proliferating cells of the yeast *Saccharomyces cerevisiae* [11-20]. Thus, *S. cerevisiae*, a unicellular eukaryote amenable to comprehensive molecular analyses [21, 22], can be used as a model organism for the discovery of selective anti-tumor small molecules that target aerobic glycolysis or lipogenesis [19, 23-32]. In this review, we compare molecular and cellular mechanisms underlying aerobic glycolysis and lipogenesis in cancer cells and rapidly proliferating fermenting yeast. Based on our analysis, we propose a novel yeast-based chemical genetic screen for anti-tumor pharmaceuticals that kill cancer cells if used at concentrations that do not compromise functionality and viability of non-cancerous cells. This high-throughput screen is aimed at the identification of small molecules capable of increasing the fatty acid desaturation index of membrane lipids in fermenting yeast cells, thereby eliciting their liponecrotic death.

## LIPONECROSIS IN YEAST: A CELL DEATH PROGRAM CAUSED BY CHANGES IN MEMBRANE LIPIDS

Our recent studies in the yeast *S. cerevisiae* have discovered and characterized a previously unknown form of programmed cell death (PCD) called “liponecrosis” [25, 33-35]. Liponecrotic PCD can be instigated by a short-term exposure of yeast to exogenous palmitoleic acid (POA), a 16-carbon monounsaturated fatty acid (16:1 n-7) [25]. Yeast cells undergoing liponecrotic PCD do not display morphological and biochemical hallmarks of the well-characterized apoptotic, autophagic or regulated necrotic forms of PCD. Indeed, unlike cell commitment to apoptotic PCD known to be accompanied by fragmentation of the nucleus and externalization of phosphatidylserine (PS) within the plasma membrane (PM) bilayer [36, 37], the commitment of yeast to the liponecrotic form of PCD does not involve nuclear fragmentation or PS enrichment in the extracellular (outer) leaflet of the PM [25, 35]. Furthermore, in contrast to cells undergoing autophagic PCD and therefore accumulating an excessive number of double-membraned vesicles called autophagosomes [36, 38-40], yeast cells that undergo liponecrotic PCD do not display such vast autophagic vacuolization of the cytoplasm [35]. Moreover, contrary to cells undergoing regulated necrotic PCD, which is characterized by a clearly visible rupture of the PM [41-44], yeast cells committed to liponecrotic PCD do not exhibit any noticeable perforations in the PM [35]. However, the necrotic and liponecrotic forms of PCD share at least one common trait - i.e., a substantial rise in the permeability of the PM for propidium iodide (PI) and other small molecules [25, 35, 41, 43, 44].

**Figure 1:**
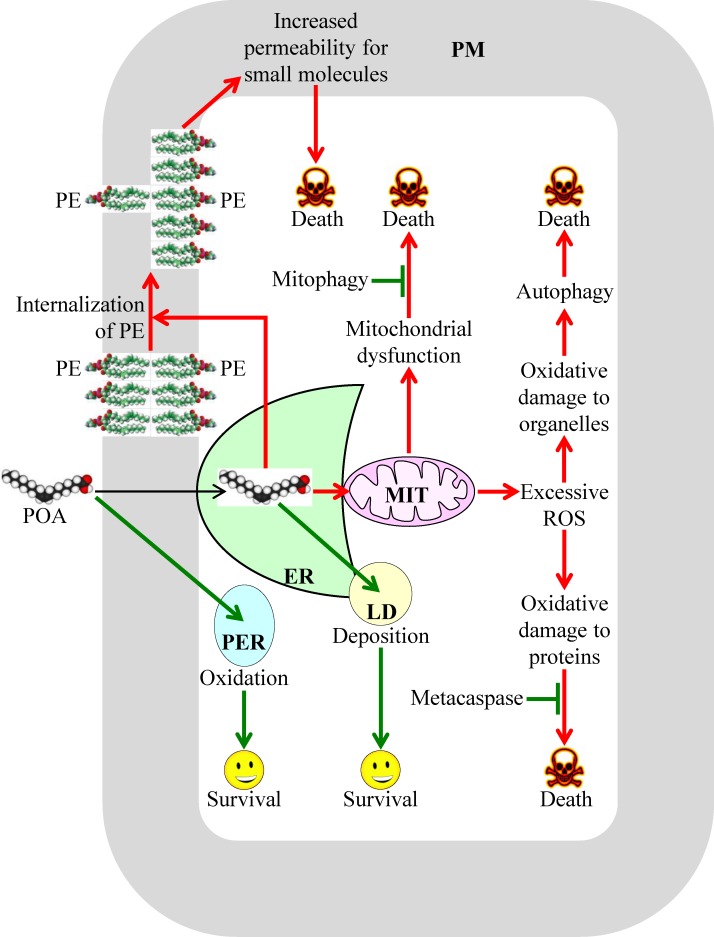
A model for the molecular mechanism underlying a liponecrotic form of programmed cell death (PCD) in yeast A brief exposure of yeast to exogenously added palmitoleic acid (POA) can trigger liponecrotic PCD, which differs from all other presently known programs of cell death. Liponecrosis in yeast is caused by a massive remodeling of lipid metabolism and lipid transport in the endoplasmic reticulum (ER), peroxisomes (PER), plasma membrane (PM), lipid droplets (LD) and mitochondria (MIT). Activation arrows and inhibition bars denote pro-death cellular processes (which are displayed in red color) or pro-survival cellular processes (which are displayed in green color). See text for more details. Abbreviations: PE, phosphatidylethanolamine; ROS, reactive oxygen species.

The molecular mechanism underlying liponecrosis has begun to emerge; it is driven by an extensive remodeling of lipid metabolism and lipid transport in yeast cells briefly exposed to exogenous POA [34, 35]. A model for such mechanism is depicted schematically in Figure 1. The model posits that the extent of yeast susceptibility to liponecrotic PCD depends on the relative rates of pro-death and pro-survival cellular processes. In Figure 1 these processes are displayed in red or green color, respectively.

The pro-death cellular processes accelerating liponecrotic PCD can create the acute cellular stress. These processes are elicited when exogenously added POA is initially used for the synthesis of POA-containing phospholipids in the endoplasmic reticulum (ER); the bulk quantities of these phospholipids then accumulate in the membrane bilayers of mitochondria and PM [34, 35], likely after being transported from the ER to these membrane bilayers *via* mitochondria-ER and PM-ER junctions (Figure 1) [45-48]. The buildup of POA-containing phospholipids in the inner and outer mitochondrial membranes of yeast committed to liponecrosis compromises mitochondrial functionality because it deteriorates such vital mitochondrial processes as respiration, electrochemical membrane potential and ATP synthesis [34, 35]. These dysfunctional mitochondria are unable to generate ATP in quantities that are sufficient for the energy-demanding process of assimilating exogenously added POA into triacylglycerols (TAG); TAG are neutral lipids initially synthesized in the ER and then deposited in lipid droplets (LD) [34, 35]. The synthesis and deposition of POA-containing TAG are considered pro-survival processes because they allow a reduction in the incorporation of POA into phospholipids, thus lowering their accumulation in the membranes of the ER, mitochondria and PM (Figure 1) [34, 49-52]. The dysfunctional mitochondria that are formed in yeast cells committed to liponecrosis can be selectively eliminated in the process of mitophagy. This autophagic degradation of dysfunctional mitochondria operates as a pro-survival process [34, 35], probably because of its well-known essential role in sustaining a population of functional mitochondria in a yeast cell (Figure 1) [53-55].

The accumulation of POA-containing phospholipids in both mitochondrial membranes can commit yeast to liponecrotic PCD not only because it weakens mitochondrial respiration, membrane potential and ATP synthesis but also because it considerably enhances the formation of reactive oxygen species (ROS) in mitochondria [34]. Due to such formation of mitochondrial ROS in bulk quantities, the cellular concentrations of ROS outside mitochondria in yeast committed to liponecrosis can exceed a cytotoxic threshold. This not only considerably reduces functionalities of various organelles by oxidatively damaging their protein and lipid constituents but also compromises cellular proteostasis by oxidatively damaging protein molecules confined to the cytosol (Figure 1) [34]. The numerous oxidatively damaged and dysfunctional organelles accumulated in yeast committed to liponecrosis undergo massive degradation. This pro-death process is executed by the cytosolic serine/threonine protein kinase Atg1 and several other proteins known to govern a non-selective autophagic breakdown of various organelles (Figure 1) [34, 35, 56, 57]. The oxidatively damaged, dysfunctional, unfolded and aggregated cytosolic proteins that amass in yeast committed to liponecrosis are degraded in a pro-survival process executed by the metacaspase Yca1 and serine protease Nma111 (Figure 1) [34]. Of note, Yca1 and Nma111 are known to be the key components of the caspase-dependent apoptotic pathway for breakdown of cytosolic proteins in yeast undergoing an apoptotic mode of PCD [58-60].

The accumulation of POA-containing phospholipids in the PM of yeast committed to liponecrosis leads to a re-distribution of phosphatidylethanolamine (PE) from the extracellular (outer) leaflet to the intracellular (inner) leaflet of the PM. The resulting depletion of PE in the outer leaflet of the PM is a pro-death process because it substantially increases the permeability of the PM for PI and other small molecules (Figure 1) [25, 34]. Such re-distribution of PE within the PM bilayer is driven by the alkaline-pH- and lipid-asymmetry-responsive Rim101 signaling pathway, which can be activated in response to the buildup of POA-containing phospholipids in this membrane bilayer [34]. Noteworthy, the Rim101 signaling pathway has been shown not only to accelerate the movement of PE from the outer leaflet of the PM to its inner leaflet but also to decelerate the movement of this phospholipid across the PM bilayer in the opposite direction [61-63].

It needs to be emphasized that at least two cellular processes can decelerate liponecrotic PCD because they both prevent a portion of exogenously added POA from being used for the synthesis of POA-containing phospholipids. As previously mentioned, one of these pro-survival cellular processes is POA incorporation into TAG and the ensuing deposition of these neutral lipids in LD (Figure 1) [34, 35]. Another such pro-survival cellular process is POA oxidation in peroxisomes of yeast cells exposed to this monounsaturated fatty acid (Figure 1) [34, 35]; peroxisomes are known for the essential role they play in oxidative degradation of fatty acids [64, 65].

## AEROBIC GLYCOLYSIS AND LIPOGENESIS: SIMILAR METABOLIC FEATURES OF THE FERMENTING YEAST *S. CEREVISIAE* AND CANCER CELLS

A body of evidence implies that rapidly proliferating cells of the yeast *S. cerevisiae* grown in nutrient- and glucose-rich media exhibit some metabolic features that are similar to the key tumorigenic metabolic traits of different types of cancer cells [15, 17, 19, 20, 32, 66-68].

**Figure 2:**
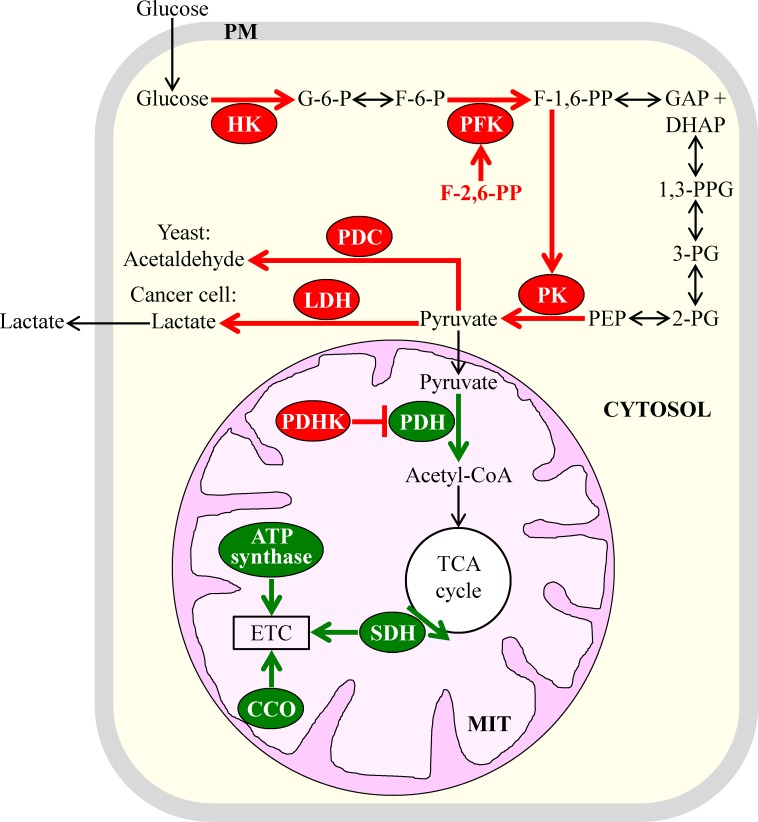
Some of the key metabolic processes underlying aerobic glycolysis in the fermenting yeast *S. cerevisiae* and cancer cells have similar rates and patterns of regulation in these two cell types Because of these similarities, aerobic glycolysis is a common metabolic feature of rapidly proliferating yeast and cancer cells. Enzymes, metabolites and processes whose activities, concentrations and rates are increased or decreased are displayed in red or green color, respectively. See text for more details. Abbreviations: CCO, cytochrome *c* oxidase; ETC, electron transport chain; HK, hexokinase; PDC, pyruvate decarboxylase; PDH, pyruvate dehydrogenase; PDHK, pyruvate dehydrogenase kinase; PFK, phosphofructokinase; PK, pyruvate kinase; PM, plasma membrane; LDH, lactate dehydrogenase; MIT, mitochondrion; SDH, succinate dehydrogenase; TCA, tricarboxylic acid.

One of these similar metabolic traits is aerobic glycolysis, also known as the Warburg effect. This trait consists in the ability of cancer cells to metabolically convert glucose to lactate under aerobic conditions [18, 20, 69-71]. In all cancer cell types, this ability is known to be caused by enhanced glucose uptake and glycolysis, and in many types of cancer cells, such ability is also due to “the Crabtree effect” of suppressing mitochondrial respiration and oxidative phosphorylation [72-76]. It should be stressed that the conversion of glucose to lactate taking place in cancer cells under aerobic conditions is substantially less efficient in terms of ATP production per molecule of glucose than the one observed in non-cancerous cells under the same conditions [12, 20, 72, 77, 78]. In the presence of oxygen, these non-cancerous cells use the glycolytic metabolic pathway to convert glucose to pyruvate, which is then metabolized to carbon dioxide via oxidative phosphorylation in mitochondria [79-83]. Several metabolic processes are known to underlie the phenomenon of aerobic glycolysis. It seems that aerobic glycolysis is common to the fermenting yeast *S. cerevisiae* and different types of cancer cells because many of these metabolic processes have similar relative rates, exhibit similar regulation patterns, and are catalyzed by enzymes sharing significant sequence homologies in yeast and cancer cells [15, 18, 66-68, 72, 77, 83]. Such similarities between the key metabolic processes underlying aerobic glycolysis in rapidly proliferating yeast and cancer cells are outlined schematically in Figure 2 and include the following: (1) elevated cytosolic levels of hexokinase isoforms that have high affinities for glucose and low sensitivities to feedback inhibition by glucose-6-phosphate [11, 13, 14, 84, 85]; (2) increased enzymatic activities of cytosolic phosphofructokinase and augmented concentrations of fructose-2,6-bisphosphate, a potent allosteric activator of phosphofructokinase [15, 18, 66, 82, 86]; (3) raised levels of cytosolic pyruvate kinase isoforms highly sensitive to allosteric activation by fructose-1,6-bisphosphate, a product of the reaction catalyzed by phosphofructokinase [82, 87-92]; (4) elevated cytosolic levels and enzymatic activities of pyruvate decarboxylase in yeast and lactate dehydrogenase in cancer cells, both of which lower mitochondrial oxidation of pyruvate by catalyzing its conversion into cytosolic acetaldehyde or cytosolic lactate, respectively [66, 82, 93-96]; (5) increased levels of mitochondrial pyruvate dehydrogenase kinase, which causes a decrease in mitochondrial pyruvate oxidation by phosphorylating and inhibiting pyruvate dehydrogenase in mitochondria [66, 97-101]; and (6) reduced levels and/or activities of such vital components of the mitochondrial ETC as succinate dehydrogenase (complex II; it is also an enzyme of the mitochondrial TCA cycle), cytochrome *c* oxidase (complex IV) and ATP synthase (complex V) [102-108]. Of note, although most known types of cancer cells permanently exhibit the Warburg effect of enhanced glucose uptake and intensified glycolysis, some of these cancer cell types display the Crabtree effect of suppressed mitochondrial TCA cycle, ETC and/or ATP synthesis only temporally whereas others never exhibit such an effect [15, 66, 109-113].

**Figure 3:**
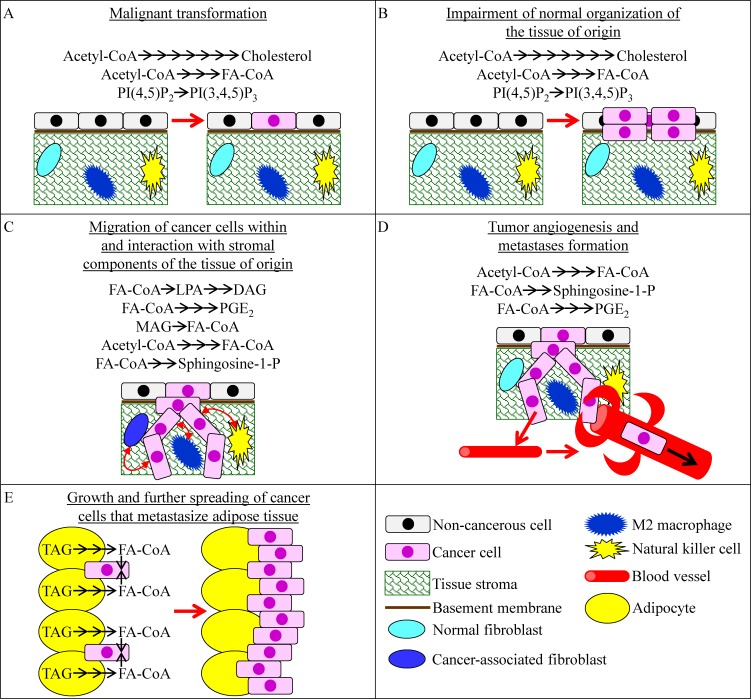
Some changes affecting lipid metabolism in cancer cells can play causal roles in certain aspects of cancer initiation, promotion and progression These aspects include malignant transformation of cells **A.**, tumorigenic impairment of normal organization of the tissue of origin **B.**, migration of cancer cells within the tissue of origin and interaction with stromal components of this tissue **C.**, tumor angiogenesis and the ensuing formation of metastases **D.**, and growth and further spreading of the cancer cells metastasizing adipose tissue **E.**. See text for more details. Abbreviations: DAG, diacylglycerol; FA-CoA, acyl-CoA ester of fatty acid; LPA, lysophosphatidic acid; MAG, monoacylglycerol; PGE_2_, prostaglandin E2; PI (4, 5) P_2_, phosphatidylinositol-4, 5-bisphosphate; PI (3, 4, 5) P_3_, phosphatidylinositol-3, 4, 5-triphosphate.

Fermenting cells of the yeast *S. cerevisiae* have another characteristic metabolic feature which is known as one of the key tumorigenic metabolic traits of cancer cells. This metabolic feature is called lipogenesis. It is common to rapidly proliferating yeast and cancer cells because both cell types require abundant quantities of membrane lipids that can be used for (1) cell growth and mitotic division, and (2) membrane trafficking and membrane-associated signaling [7, 11, 14, 16, 19, 20, 77, 80, 114-121]. Lipogenesis refers to a type of metabolic reprogramming in which the surplus metabolites produced by aerobic glycolysis can be used for the de novo synthesis of bulk quantities of membrane lipids, mainly (but not exclusively) fatty acids, phospholipids and cholesterol [1, 18, 20, 77, 114, 119]. Recent evidence indicates that certain features of such reprogramming of lipid metabolism in cancer cells may play causal roles in malignant transformation and tumor development. The features of reprogrammed lipid metabolism that can affect some specific aspects of cancer initiation, promotion and progression are recapitulated schematically in Figure 3 and include the following: (1) a stimulation of the mevalonate pathway of cholesterol synthesis, activation of fatty acid synthesis and induction of phosphatidylinositol-3,4,5-triphosphate synthesis - which can all promote malignant transformation of cells and can also contribute to the tumorigenic impairment of normal tissue organization (Figure 3A and 3B) [7, 120, 122-130]; (2) a lipogenic incorporation of fatty acids into diacylglycerol, lysophosphatidic acid and prostaglandin E_2_, as well as a lipolytic, monoacylglycerol lipase (MAGL)-driven formation of fatty acids from monoacylglycerols, all of which can help cancer cells to invade the tissue of origin by stimulating the migration of cancer cells within this tissue and also by promoting the interaction of cancer cells with stromal components of this tissue (Figure 3C) [6, 125, 131-137]; (3) the de novo synthesis of fatty acids from acetyl-CoA and the formation of lysophosphatidic acid, sphingosine-1-phosphate and prostaglandin E_2_ from fatty acids which can support cancer cell invasion within the tissue of origin by guiding the bidirectional communications between the invading cancer cells and such stromal components as cancer-associated fibroblasts, M2 macrophages and natural killer cells (Figure 3C) [125, 138-141]; (4) the de novo synthesis of fatty acids, sphingosine-1-phosphate and prostaglandin E_2_ which can accelerate tumor angiogenesis, thus facilitating the spreading of cancer cells into other tissues and supporting the formation of metastases (Figure 3D) [125, 142-148]; and (5) the lipolysis of TAG deposited in LD within adipocytes adjacent to cancer cells that metastasize adipose tissue which allows cancer cells to generate bulk quantities of fatty acids; after being transferred to metastatic cancer cells, these fatty acids can be oxidized in mitochondria to support the growth and further spreading of the cancer cells (Figure 3E) [149-152].

**Figure 4:**
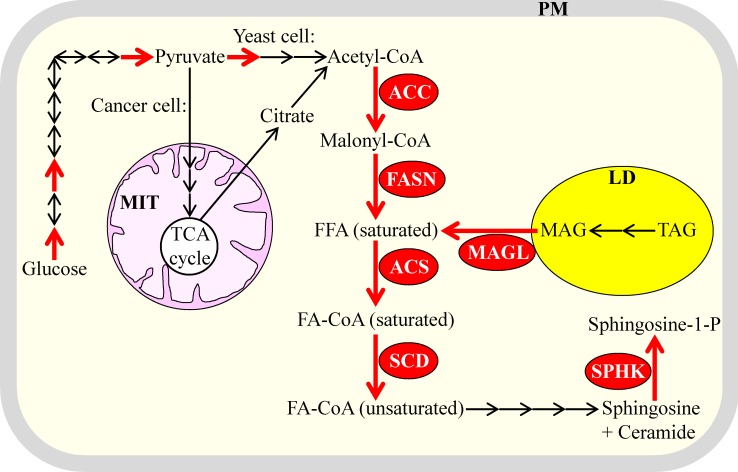
Several of the key metabolic processes underlying lipogenesis in the fermenting yeast *S. cerevisiae* and cancer cells have comparable rates and exhibit analogous patterns of regulation in these two cell types Due to such similar properties of the key metabolic processes that underlie lipogenesis, it is a common metabolic trait of the rapidly proliferating yeast and cancer cells. Enzymes and processes exhibiting increased activities and rates are displayed in red color. Glycolytic processes whose rates are elevated in yeast and cancer cells are also shown in red color; enzymes catalyzing these processes are named in Figure 2. See text for more details. Abbreviations: ACC, acetyl-CoA carboxylase; ACS, acyl-CoA synthetase; FA-CoA, acyl-CoA ester of fatty acid; FASN, fatty acid synthase; FFA, free fatty acid; LD, lipid droplets; MAG, monoacylglycerol; MAGL, monoacylglycerol lipase; MIT, mitochondria; PM, plasma membrane; SCD, stearoyl-CoA desaturase; SPHK, sphingosine-1-kinase; TAG, triacylglycerol; TCA, tricarboxylic acid.

Akin to aerobic glycolysis, lipogenesis is common to the fermenting yeast *S. cerevisiae* and cancer cells because several key processes underlying this metabolic trait exhibit comparable rates, display analogous patterns of regulation and are driven by orthologous proteins in yeast and cancer cells [1, 16, 19, 50, 51, 81, 114, 119, 120, 153, 154]. Such similar properties of the key metabolic processes underlying lipogenesis in rapidly proliferating yeast and cancer cells are displayed schematically in Figure 4 and include the following: (1) increased activities of the enzymes involved in the de novo synthesis of acyl-CoA esters of saturated fatty acids from acetyl-CoA; among these enzymes are acetyl-CoA carboxylase (ACC), fatty acid synthase (FASN) and acyl-CoA synthetase (ACS) in cancer cells and their orthologues Acc1, Fas1/Fas2 complex and Faa1 in yeast [1, 19, 155-160]; (2) an elevated enzymatic activity of stearoyl-CoA desaturase (SCD; this enzyme catalyzes the desaturation of stearoyl-CoA to oleoyl-CoA) in cancer cells and of the orthologous enzyme Ole1 in yeast [1, 5, 16, 19, 161-163]; (3) an amplified enzymatic activity of sphingosine-1-kinase (which accelerates the formation of the potent signaling molecule sphingosine-1-phosphate from acyl-CoA esters of fatty acids) in cancer cells and of its orthologues Lcb4 and Lcb5 in yeast [16, 19, 143, 164-167]; and (4) an elevated enzymatic activity of MAGL (which catalyzes the lipolytic formation of fatty acids from monoacylglycerols) in cancer cells; in rapidly proliferating yeast, the TAG lipase Tgl4 is likely to play a similar role in providing fatty acids to support cell growth, division, membrane trafficking and membrane-associated signaling [6, 19, 115, 117, 133, 137]. Noteworthy, although many types of solid tumors exhibit both aerobic glycolysis and lipogenesis, primary prostate cancers display only lipogenesis as a characteristic metabolic trait [10, 158, 168-171].

## A CURRENT APPROACH TO DEVELOPING SELECTIVE ANTI-TUMOR PHARMACEUTICALS THAT CAN REORGANIZE LIPID METABOLISM: SEARCHING FOR INHIBITORS OF LIPOGENESIS

**Figure 5:**
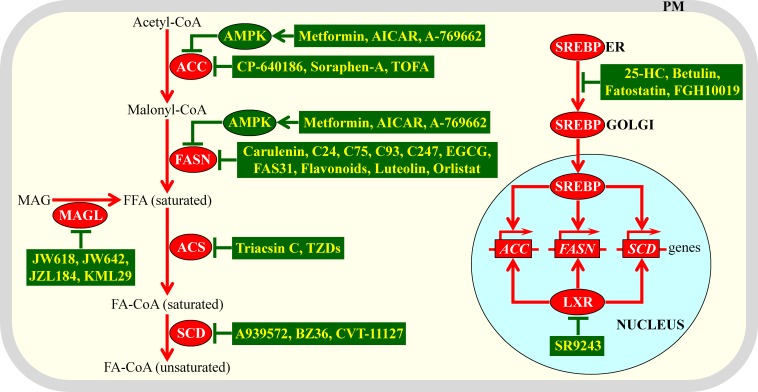
Different mechanisms by which various anti-tumor pharmaceuticals can inhibit lipogenesis in cancer cells These mechanisms decelerate lipogenesis-related processes common to rapidly proliferating yeast and cancer cells. Genes, proteins and processes whose increased expression levels, activities and rates promote tumorigenesis in cells that are not exposed to lipogenesis-inhibiting small molecules are displayed in red color. Proteins and processes whose activities and rates attenuate tumorigenesis in response to cell treatment with lipogenesis-inhibiting small molecules are displayed in green color. See text for more details. Abbreviations: 25-HC, 25-hydroxycholesterol; ACC, acetyl-CoA carboxylase; ACS, acyl-CoA synthetase; AICAR, 5-aminoimidazole-4-carboxamide ribonucleoside; AMPK, AMP-activated protein kinase; EGCG, epigallocatechin-3-gallate; ER, endoplasmic reticulum; FA-CoA, acyl-CoA ester of fatty acid; FASN, fatty acid synthase; FFA, free fatty acid; LXR, liver-X-receptor; MAG, monoacylglycerol; MAGL, monoacylglycerol lipase; PM, plasma membrane; SCD, stearoyl-CoA desaturase; SREBP, sterol regulatory element-binding protein; TOFA, 5-(tetradecyloxy)-2-furoic acid; TZDs, thiazolidinediones.

The current strategy for discovering anti-tumor pharmaceuticals that target lipid metabolism consists of searching for small molecules capable of inhibiting lipogenesis in cancer cells. Such molecules are expected to diminish the quantities of membrane lipids available to support proliferation and survival of cancer cells without impairing functionality and viability of non-cancerous cells. This strategy for uncovering anti-tumor therapeutic agents has recently been extensively exploited in a number of studies, and their major findings have been reviewed elsewhere [1-3, 6, 10, 19, 81, 82, 119, 133, 148, 155, 158, 170, 172-177]. Figure 5 schematically summarizes four different mechanisms through which various pharmacological interventions can inhibit lipogenesis in cancer cells by slowing down lipogenesis-related processes that are also known to support rapid proliferation of fermenting yeast. These mechanisms include the following: (1) a direct chemical inhibition of ACC, FASN, ACS, SCD or MAGL by a distinct set of small molecules, each of which can specifically bind to one of these enzymes (Figure 5) [1, 6, 10, 133, 155, 160, 178-191]; (2) a metformin-, 5-aminoimidazole-4-carboxamide ribonucleoside (AICAR)- or A-769662-dependent activation of AMP-activated protein kinase (AMPK), which then phosphorylates and inhibits ACC and FASN (Figure 5) [1, 10, 19, 192-197]; (3) an interaction of the small molecule SR9243 with the nuclear liver-X-receptor (LXR); such interaction attenuates the LXR-driven transcription of nuclear genes encoding ACC, FSN and SCD (Figure 5) [2, 198]; and (4) an inhibition of the ER-to-Golgi transport of sterol regulatory element-binding proteins (SREBP) by betulin and several other small molecules; such inhibition impairs the proteolytic processing of SREBP in the Golgi, thus preventing the import of active fragments of SREBP into the nucleus and attenuating SREBP-dependent transcription of nuclear genes encoding ACC, FSN and SCD (Figure 5) [1, 199-201].

## A NOVEL APPROACH TO DISCOVERING SELECTIVE ANTI-TUMOR PHARMACEUTICALS THAT CAN ALTER LIPID METABOLISM: THE SEARCH FOR ACTIVATORS OF LIPONECROSIS

A growing body of evidence indicates that the proliferation of cancer cells can be decelerated and their survival can be compromised by genetic and pharmacological interventions capable of altering (i.e. decreasing or increasing) “the fatty acid desaturation index”. The index is defined as the ratio of monounsaturated fatty acids (MUFA) to saturated fatty acids (SFA) [202, 203]. The value of this index in human cells depends mainly on the enzymatic activity of the SCD1 isoform of stearoyl-CoA desaturase. This ER-associated acyl-CoA delta-9 desaturase accelerates the introduction of a cis-double bond between carbons 9 and 10 of acyl-CoA esters of palmitic or stearic acid to produce acyl-CoA esters of palmitoleic or oleic acid, respectively [204-207]. Palmitic and stearic acid are the most abundant SFA in cancer cells, whereas palmitoleic and oleic acid are the major MUFA [5, 208-211]. All four of these fatty acids are the predominant forms of cellular lipids that can be found as free SFA or MUFA and their acyl-CoA esters. The bulk of these fatty acids (especially MUFA) in cancer cells can also be incorporated into membrane phospholipids, neutral lipids and sphingolipids [114, 158, 208-211].

**Figure 6:**
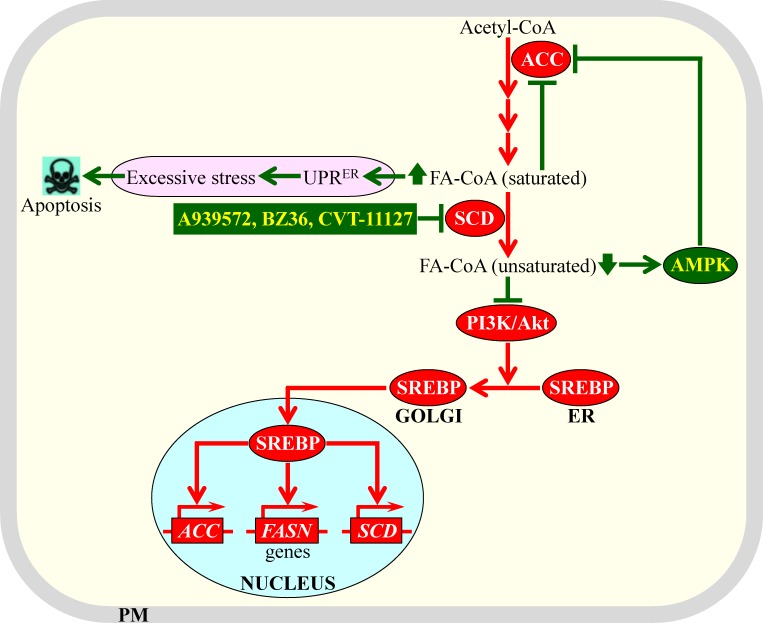
Mechanisms through which an inhibition of stearoyl-CoA desaturase (SCD) by some pharmaceuticals can cause potent anti-tumor effects Such inhibition elicits a rise of cellular concentrations of acyl-CoA esters of saturated fatty acids and a concomitant decline of cellular concentrations of acyl-CoA esters of unsaturated fatty acids. Genes, proteins and processes whose increased expression levels, activities and rates promote tumorigenesis in cells that are not exposed to the SCD inhibitors are displayed in red color. Proteins and processes whose activities and rates attenuate tumorigenesis in response to cell treatment with the SCD inhibitors are displayed in green color. See text for more details. Abbreviations: ACC, acetyl-CoA carboxylase; AMPK, AMP-activated protein kinase; Akt, a serine/threonine-specific protein kinase; ER, endoplasmic reticulum; FA-CoA, acyl-CoA ester of fatty acid; FASN, fatty acid synthase; PI3K/Akt, phosphatidylinositol-3 kinase/Akt signaling pathway; PM, plasma membrane; SREBP, sterol regulatory element-binding protein; UPR^ER^, the unfolded protein response signaling pathway in the ER.

Inhibition of SCD1 activity, either by certain genetic manipulations or by some pharmaceuticals (see Figure 5), decreases the fatty acid desaturation index. Such inhibition has been shown to exhibit potent anti-tumor effects in different forms of cancer by affecting various aspects of cancer initiation, promotion and progression [5, 163, 180, 184, 186, 188, 212-219]. Recent studies have suggested two different types of mechanisms through which such inhibition of SCD1 and the resulting decrease of the fatty acid desaturation index (i.e. a rise of cellular SFA concentration and a concomitant decline of cellular MUFA concentration) can cause the observed anti-tumor effects. These two types of mechanisms are recapitulated schematically in Figure 6. They include the following: (1) the build-up of SFA alters the structure of the ER membrane and impairs its functionality, thereby eliciting the unfolded protein response signaling pathway in the ER and excessively stressing this organelle [162, 220-229]; if such SFA-driven stress in the ER exceeds a cytotoxic threshold in cancer cells with inhibited SCD1, these cells undergo apoptosis [215, 219]; and (2) SCD1 inhibition in cancer cells substantially decelerates the flow of the surplus glycolytic metabolites into fatty acid synthesis (and thus, suppresses lipogenesis) by eliciting a significant decrease in the enzymatic activity of ACC; this suppressing effect of SCD1 inhibition on lipogenesis is due to the following demonstrated abilities of such inhibition: (a) it activates AMPK, which then phosphorylates and inhibits ACC, (b) it elevates cellular concentrations of saturated acyl-CoA species known to inhibit ACC allosterically, and (c) it attenuates the phosphatidylinositol-3 kinase/Akt signaling pathway needed for SREBP-driven transcription of nuclear genes encoding ACC and other lipogenic enzymes [5, 80, 163, 180, 188, 214, 230-233].

Noteworthy, various cell lines of breast, colorectal and prostate cancers have been shown to possess substantially increased lipogenic activities [217]. Moreover, in primary prostate cancers, lipogenesis (but not aerobic glycolysis) is known to be the only prominent tumorigenic trait of metabolism [10, 158, 168-171]. It needs to be emphasized that cells of all these highly lipogenic cancer forms have been shown to exhibit a considerably reduced fatty acid desaturation index (i.e. the concentrations of MUFA in these cells are decreased whereas cellular concentrations of SFA are increased) if the cells are not exposed to an anti-tumor agent [217]. However, these highly lipogenic cancer cells display a substantially elevated fatty acid desaturation index (i.e. the concentrations of both SFA and MUFA in these cells are decreased whereas cellular concentrations of polyunsaturated (PUFA) fatty acids are substantially increased) if the cells are subjected to genetic or pharmaceutical interventions lowering lipogenesis and causing robust anti-tumor effects [217]. Because the highly lipogenic cells of breast, colorectal and prostate cancers exposed to such anti-tumor interventions exhibited an elevated fatty acid desaturation index and increased concentrations of peroxidation-susceptible PUFA, these cells displayed raised concentrations of oxidatively damaged membrane lipids, enhanced susceptibility to oxidative stress, declined protein mobility within lipid membrane bilayers and augmented permeability of the PM bilayer to small molecules [217]. It is conceivable therefore that some small molecules capable of increasing the fatty acid desaturation index in the highly lipogenic cancer cells (for instance, by stimulating SCD1 activity) may exhibit robust anti-tumor effects in these rapidly proliferating cells. Because lipogenesis is a common metabolic trait of cancer cells and rapidly proliferating fermenting yeast (see above), it is likely that such anti-tumor small molecules may also cause rapid loss of cell viability in yeast.

Furthermore, it has been demonstrated that cancer cells constitutively overexpressing Ole1, a yeast ortholog of human SCD1, exhibit an increased fatty acid desaturation index due to the elevated concentrations of MUFA in these cells [234, 235]. It should be stressed that such a rise in the fatty acid desaturation index not only enhances the fluidity of membrane bilayers in these cancer cells but also considerably increases cell susceptibility to exogenous tumor necrosis factor [234, 235]. Of note, an elevated level of Ole1 expression has been shown to impair growth and division of fermenting yeast [236]. Thus, again, it seems that pharmaceuticals capable of increasing the fatty acid desaturation index in rapidly proliferating cancer cells by elevating SCD activity may have high anti-tumor potential. It is feasible that these anti-tumor pharmaceuticals may also compromise survival of rapidly proliferating yeast.

Moreover, a yeast mutant concurrently lacking acyltransferases Lro1, Dga1, Are1 and Are2 is known to be unable to incorporate acyl-CoA esters of MUFA into TAG [237]. It has been shown that cells of this mutant exhibit excessive proliferation of ER membranes and rapidly die only if exposed to exogenous MUFA [237]. In contrast, an exposure of these mutant cells to SFA does not cause ER membrane proliferation or loss of cell viability [237]. It is therefore likely that some small molecules capable of reducing the incorporation of MUFA into TAG may kill fermenting yeast cells. Due to similarities of lipogenic processes taking place in these rapidly proliferating cells and in cancer cells (see above), it is conceivable that such small molecules may also exhibit robust anti-tumor effects.

**Figure 7:**
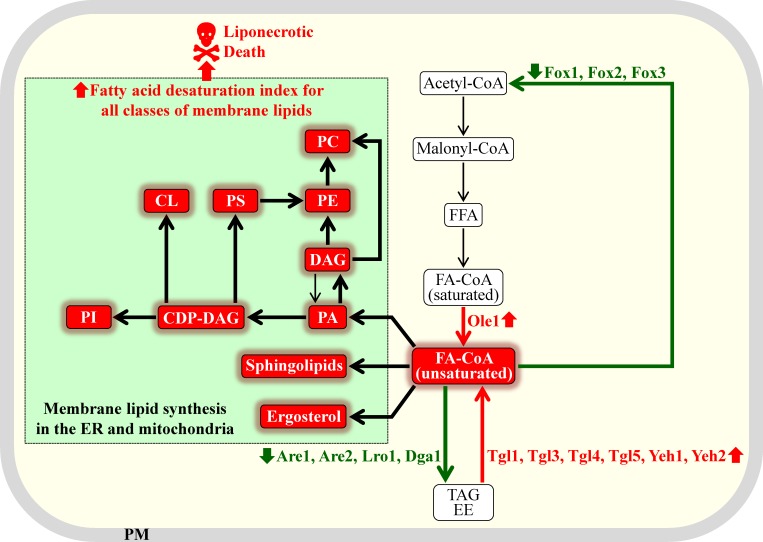
Different ways of increasing the fatty acid desaturation index of various membrane lipids in yeast with the help of small molecules A small molecule eliciting any of these methods for increasing the fatty acid desaturation index can commit rapidly proliferating fermenting yeast to liponecrotic programmed cell death. Proteins and processes whose elevated or lowered activities and rates can increase the fatty acid desaturation index of membrane lipids are displayed in red or green color, respectively. Thick black arrows indicate processes whose rates are expected to be intensified in yeast exposed to a small molecule capable of increasing the fatty acid desaturation indexes of various membrane lipids. See text for more details. Abbreviations: CDP-DAG, cytidine diphosphate diacylglycerol; CL, cardiolipin; DAG, diacylglycerol; EE, ergosteryl ester; ER, endoplasmic reticulum; FA-CoA, acyl-CoA ester of fatty acid; FFA, free fatty acid; PA, phosphatidic acid; PC, phosphatidylcholine; PE, phosphatidylethanolamine; PI, phosphatidylinositol; PM, plasma membrane; PS, phosphatidylserine; TAG, triacylglycerol.

Based on the above findings, we propose the following approach to using fermenting yeast as a model organism for the discovery of anti-tumor pharmaceuticals that can alter lipid metabolism. As discussed above, small molecules that can increase the fatty acid desaturation index of free fatty acids, phospholipids, ergosterol and sphingolipids in fermenting yeast cells may not only cause death of these cells but may also selectively kill rapidly proliferating cancer cells. There are at least four different ways of increasing the fatty acid desaturation index of various lipid classes in yeast with the help of small molecules. These ways are outlined schematically in Figure 7 and include the following: (1) elevating the enzymatic activity of the delta-9 fatty acid desaturase Ole1, which catalyzes the formation of acyl-CoA esters of unsaturated fatty acids from acyl-CoA esters of saturated fatty acids [16, 50, 51]; (2) raising the enzymatic activities of lipases involved in the hydrolytic formation of acyl-CoA esters of unsaturated fatty acids from TAG and ergosteryl esters (EE); these lipases include Tgl1, Tgl3, Tgl4, Tgl5, Yeh1 and Yeh2 [16, 50, 51]; (3) decreasing the enzymatic activities of acyltransferases that accelerate the incorporation of acyl-CoA esters of unsaturated fatty acids into TAG and EE; among these acyltransferases are Dga1, Lro1, Are1 and Are2 [16, 50, 51]; and (4) decreasing the activities of peroxisomal enzymes Fox1, Fox2 and Fox3, all of which are involved in degradative β-oxidation of acyl-CoA esters of unsaturated fatty acids [16, 50, 51]. It needs to be emphasized that a small molecule eliciting any of these methods for increasing the fatty acid desaturation index of membrane lipids is expected to cause a buildup of unsaturated lipids in the membrane bilayers of ER, mitochondria and PM. As it has been mentioned in the first section of this review, the excessive accumulation of POA-containing lipids in the membrane bilayers of ER, mitochondria and PM can commit yeast to liponecrotic PCD; POA is a 16-carbon MUFA [25, 33-35]. It is therefore conceivable that pharmaceuticals causing an increase in the fatty acid desaturation index of membrane lipids can trigger liponecrotic cell death of fermenting yeast. As discussed above, due to the similarities of lipogenic processes taking place in rapidly proliferating fermenting yeast and in cancer cells, it is plausible that such pharmaceuticals may also have robust anti-tumor effects.

Of note, our recent studies have convincingly demonstrated the applicability of yeast as a model organism for the discovery of selective anti-tumor small molecules targeting a certain aspect of tumorigenesis. Specifically, our high-throughput chemical genetic screen for pharmaceuticals that can extend yeast longevity has identified lithocholic acid (LCA), the most hydrophobic bile acid, as one of them [25]. We have uncovered the molecular and cellular mechanisms through which LCA increases the lifespan of chronologically aging yeast [33, 45, 55, 238-241]. It appears that LCA is not only a longevity-extending molecule in yeast but also a potent anti-tumor agent in human cells. Indeed, at concentrations that are not cytotoxic to non-cancerous cells, LCA can selectively kill cultured human breast, prostate and neuroblastoma cancer cells [26, 238, 242]. Of note, cancer is considered a disease of aging [7, 243-246]. This assertion is based on the following findings: (1) age in the major risk factor for developing cancer; (2) genetic, dietary and pharmacological interventions that delay aging in animal models reduce the incidence of cancer; and (3) some of the evolutionarily conserved pathways and mechanisms underlying cancer and aging are common to these two inherently complex biological phenomena [7, 243, 247-251].

**Figure 8:**
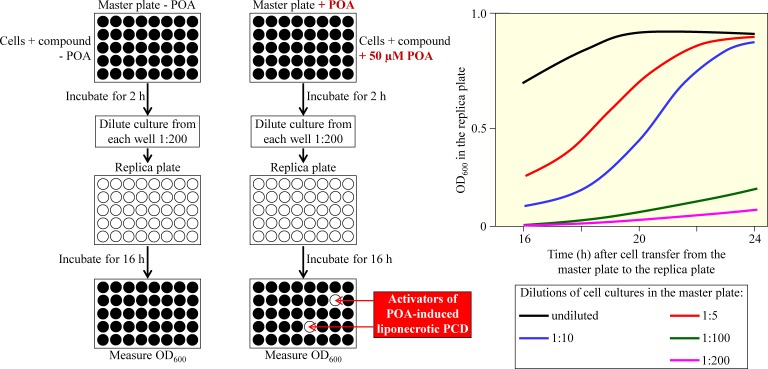
A microplate assay for measuring the viability of yeast cells by monitoring the optical density of a yeast culture at 600 nm (OD_600_) This high-throughput assay can be used to screen compound libraries for small molecules that activate palmitoleic acid (POA)-induced liponecrotic cell death of fermenting yeast. Fast growing yeast cells are first incubated for 2 h in master microplates containing growth medium and various small molecules, with or without POA. A small aliquot of each culture is then diluted in an individual well of a replica microplate supplemented with growth medium only. The replica microplate is incubated for 16 h, and the OD_600_ of a cell culture in each well is measured. For a cell culture in the replica microplate incubated for 16 h after cell transfer from the master microplate, the value of OD_600_ is directly proportional to the number of viable cells in a well of the replica microplate. See text for more details. Abbreviation: PCD, programmed cell death.

We therefore propose here to use a high-throughput screen for small molecules that can activate liponecrotic PCD of fermenting yeast by increasing the fatty acid desaturation index of membrane lipids. This screen is depicted schematically in Figure 8. It is based on employing a microplate assay for measuring the viability of yeast cells *via* monitoring the optical density of a yeast culture at 600 nm (OD_600_). In the proposed screen, fast proliferating yeast cells cultured in a nutrient- and glucose-rich medium and progressing through the exponential growth phase are initially incubated for 2 h in master microplates. Yeast cultures in individual wells of these master microplates are supplemented with various small molecules from a compound library. Wells in one set of master microplates do not contain POA, whereas wells in another set of such microplates contain 50 μM POA. At this concentration, exogenously added POA kills not more than 20% of the total number of cells in a yeast culture [34, 35]. After 2 h of incubation in master microplates, a small aliquot of each culture is diluted 1:200 in an individual well of a replica microplate. Wells in all replica microplates contain only a growth medium rich in nutrients and glucose. After 16 h of incubation, the OD_600_ of a cell culture in each well of the replica microplates is measured. This incubation time is optimal for the value of OD_600_ to correlate with the number of viable cells in a well of the replica microplate (Figure 8) [25]. The small molecules from a compound library that cause the highest extent of decrease in the value of the OD_600_ of a cell culture are chosen as “lead” compounds. These compounds are then used for “cherry-picking” of “lead” compounds capable of activating liponecrotic PCD of yeast by increasing the fatty acid desaturation index of membrane lipids.

The high-throughput screen outlined in Figure 8 can be conducted using the following yeast strains: (1) a wild-type strain, which exhibits “normal” concentrations and activities of Ole1, Tgl1, Tgl3, Tgl4, Tgl5, Yeh1, Yeh2, Dga1, Lro1, Are1 and Are2; as outlined above, activities of all these enzymes play essential roles in defining the intracellular concentrations of acyl-CoA esters of MUFA (Figure 7); (2) a yeast strain constitutively overexpressing the delta-9 fatty acid desaturase Ole1, which catalyzes the formation of acyl-CoA esters of MUFA (Figure 7); as mentioned above, this mutant strain exhibits an increased fatty acid desaturation index of all membrane lipid classes because it accumulates MUFA at high concentrations [234, 235]; and (3) a yeast strain concurrently lacking acyltransferases Lro1, Dga1, Are1 and Are2; as outlined earlier in the text, this strain is unable to incorporate acyl-CoA esters of MUFA into TAG (Figure 7) and, thus, amasses MUFA-containing membrane lipids at high concentrations [237]. It is possible that the above high-throughput screen may reveal small molecules causing a substantial decrease in the OD_600_ of a cell culture not only for the yeast strain overexpressing Ole1, but also for the yeast strain concurrently lacking acyltransferases Lro1, Dga1, Are1 and Are2. Moreover, among such small molecules there may be those that do not decrease (or decrease only slightly) the OD_600_ of a cell culture for a wild-type strain of yeast. These small molecules are expected to exhibit a combination of the following two features: (1) they may cause rapid loss of cell viability only in yeast strain exhibiting increased concentrations of acyl-CoA esters of POA (and of other MUFA) and elevated concentrations of POA-containing (and of other MUFA-containing) membrane lipids; and (2) they may selectively kill only lipogenic cancer cells without impairing functionality and viability of non-cancerous cells. The small molecules exhibiting a combination of these two features have potential to be selective anti-tumor therapeutic agents that cause liponecrotic death of cancer cells, while sparing non-cancerous cells.

A compound library which can be used for the high-throughput screen outlined in Figure 8 may include many small molecules that are currently known to act as follows: (1) to be target-specific activators of Ole1, Tgl1, Tgl3, Tgl4, Tgl5, Yeh1, Yeh2, Dga1, Lro1, Are1 or Are2 in fermenting yeast; and/or (2) to be target-specific inhibitors of Dga1, Are1, Are2, Fox1, Fox2 or Fox3 in fermenting yeast. These target-specific small molecules have been revealed in numerous studies on yeast chemogenomic profiling that have been conducted with the help of haploinsufficiency profiling, homozygous deletion profiling and multicopy gene suppression profiling assays in a high-throughput, genome-wide format [27-29, 31, 252-256].

## CONCLUSIONS

Lipogenesis is a characteristic rewiring of lipid metabolism in cells of various cancers. It consists in channeling the excess metabolites made during aerobic glycolysis into the de novo synthesis of membrane lipids in large quantities. Lipogenesis is one of the key tumorigenic features of cancer cell metabolism, and it is known to play causal roles in certain aspects of cancer initiation, promotion and progression. Recent evidence indicates that lipogenesis is common to cancer cells and rapidly proliferating cells of the yeast *S. cerevisiae*, perhaps because several key processes underlying lipogenesis in these cell types have similar relative rates, exhibit comparable regulation patterns and depend on orthologous proteins. The presently used strategy for the discovery of potential anti-tumor small molecules consists of uncovering pharmaceuticals that inhibit lipogenesis in cancer cells. Emergent evidence suggests that chemical compounds capable of increasing the fatty acid desaturation index of membrane lipids can elicit liponecrotic cell death of both cancer cells and fermenting yeast. This creates an opportunity to use rapidly proliferating yeast as a model organism for a high-throughput screen aimed at the identification of such compounds. The chemical genetic screen proposed here has the potential to develop selective anti-tumor pharmaceuticals that cause liponecrotic death of cancer cells but do not affect functionality and viability of non-cancerous cells.
